# Proprioceptors in extraocular muscles

**DOI:** 10.1113/EP090765

**Published:** 2023-03-03

**Authors:** Roland Blumer, Génova Carrero‐Rojas, Paula M. Calvo, Johannes Streicher, Rosa R. de la Cruz, Angel M. Pastor

**Affiliations:** ^1^ Center of Anatomy and Cell Biology, Division of Anatomy, Medical Image Cluster Medical University Vienna Vienna Austria; ^2^ Departamento de Fisiología, Facultad de Biología Universidad de Sevilla Sevilla Spain; ^3^ Department of Anatomy and Biomechanics, Division of Anatomy and Developmental Biology Karl Landsteiner University of Health Science Krems an der Donau Austria

**Keywords:** eye muscle, Golgi tendon organs, muscle spindles, palisade endings, proprioception

## Abstract

Proprioception is the sense that lets us perceive the location, movement and action of the body parts. The proprioceptive apparatus includes specialized sense organs (proprioceptors) which are embedded in the skeletal muscles. The eyeballs are moved by six pairs of eye muscles and binocular vision depends on fine‐tuned coordination of the optical axes of both eyes. Although experimental studies indicate that the brain has access to eye position information, both classical proprioceptors (muscle spindles and Golgi tendon organ) are absent in the extraocular muscles of most mammalian species. This paradox of monitoring extraocular muscle activity in the absence of typical proprioceptors seemed to be resolved when a particular nerve specialization (the palisade ending) was detected in the extraocular muscles of mammals. In fact, for decades there was consensus that palisade endings were sensory structures that provide eye position information. The sensory function was called into question when recent studies revealed the molecular phenotype and the origin of palisade endings. Today we are faced with the fact that palisade endings exhibit sensory as well as motor features. This review aims to evaluate the literature on extraocular muscle proprioceptors and palisade endings and to reconsider current knowledge of their structure and function.

## Abstract (highlights)

- **What is the topic of this review?**
  This review aims to evaluate the literature on proprioceptors and particular nerve specializations (palisade endings) in mammalian extraocular muscles (EOMs) and to reconsider current knowledge of their structure and function.
- **What advances does it highlight?**
  Classical proprioceptors (muscle spindles and Golgi tendon organs) are absent in the EOMs of most mammals. Instead, palisade endings are present in most mammalian EOMs. For many years, palisade endings were considered to be sensory but recent studies show that they combine sensory and motor features. The functional significance of palisade endings is still debated.

## 1 INTRODUCTION

The term proprioception was introduced by Sherrington (1907), and it is best described as a position sense that lets us be aware of the location and movement of body parts. Proprioceptive sensations arise in skeletal muscles, skin and joints and they are the foundation for coordinated movements (Bewick & Banks, 2015; Smith et al., 2020). The value of proprioception is evident in animal models with proprioceptive deficits (Chen et al., 2007) and in subjects who have lost their proprioception sense (Gallagher & Cole, 1995; Gordon et al., 1995). Without proprioception, movements are uncoordinated, and tasks like standing, reaching out for a cup of coffee or walking are impossible (Gallagher & Cole, 1995).

**FIGURE 1:**
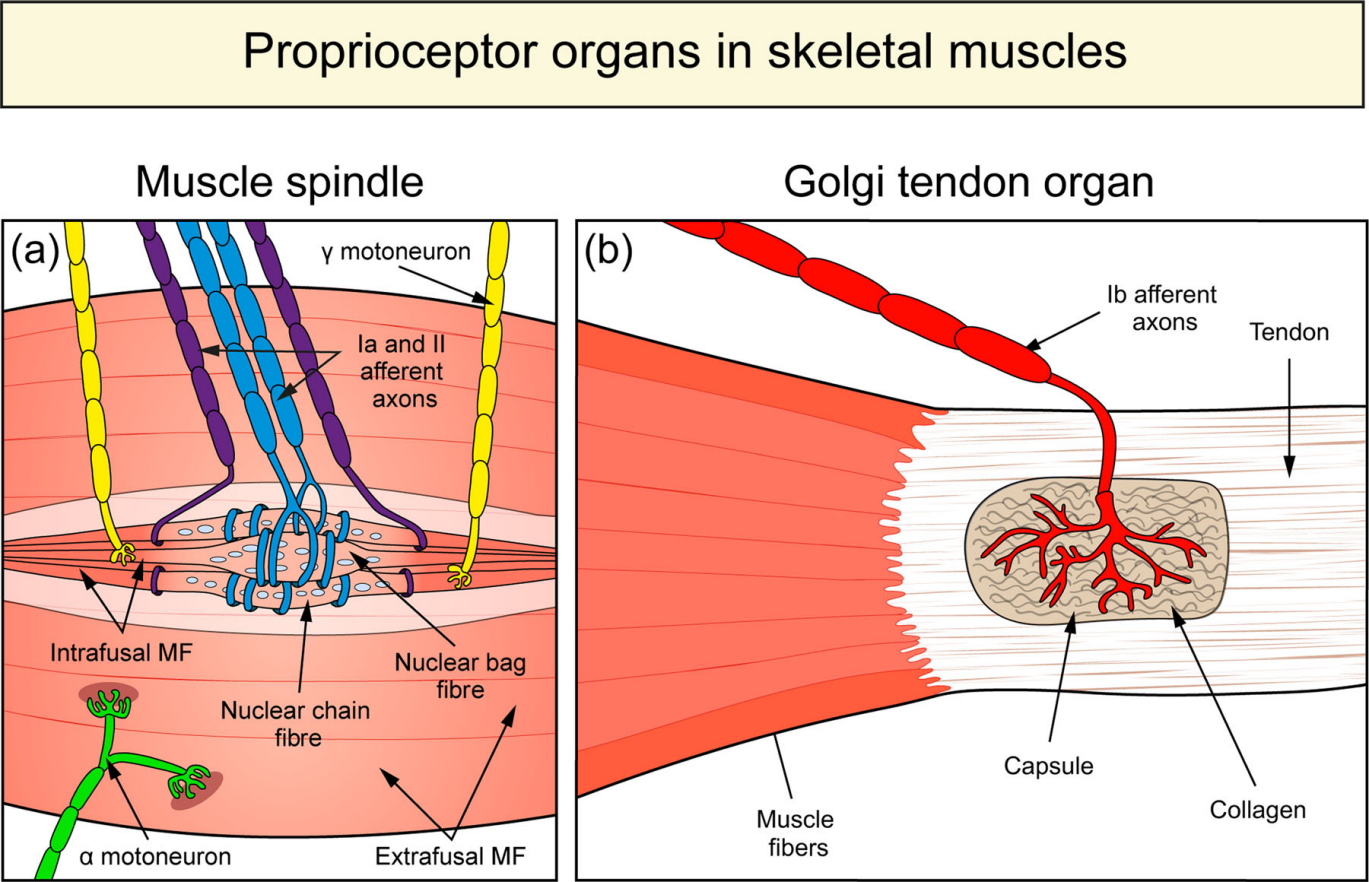
Classical proprioceptor organs in skeletal muscles of mammals. (a) Schematic drawing of a muscle spindle. The muscle spindle contains nuclear bag and nuclear chain intrafusal fibres. Both of them are innervated by Ia afferent axons (blue) that form annulospiral endings in the equatorial region. Some intrafusal muscle fibres are also innervated by II afferent axons (purple). Additionally, efferent γ‐motoneurons (yellow) establish motor contacts on the intrafusal muscle fibres in the muscle spindle's polar regions. Outside the muscle spindle, an α‐motoneuron (green) establishes synaptic contacts with the extrafusal muscle fibres. (b) Schematic drawing of a Golgi tendon organ. This organ is located at the muscle–tendon junction and is filled with collagen bundles. A single large Ib afferent axon (red) enters the Golgi tendon organ and after splitting into several branches, nerve terminals establish contact with the collagen fibres of the Golgi tendon organ.

Specialized muscle receptors, the proprioceptors, play a key role in proprioception (Bewick and Banks (2015). Two types of proprioceptors can be distinguished in mammalian skeletal muscles: muscle spindles and Golgi tendon organs (Figure 1). Muscle spindles lie deeply embedded in the muscle and are spindle‐shaped, encapsulated organs. They contain a bundle of muscle fibres (intrafusal muscle fibres) which are endowed with sensory nerve terminals in the muscle spindle's mid‐region (equatorial region). The sensory nerve terminals are stretch‐sensitive and register changes in muscle length. Fine‐tuning of the muscle spindle's sensitivity is achieved by motor innervation (γ‐motoneurons) at the spindle's pole. Unlike muscle spindles, Golgi tendon organs lie at the muscle–tendon interface. They have a capsule of perineural cells and are filled with collagen bundles and nerve terminals. Because Golgi tendon organs lie in series with the muscle fibres, they register muscle contraction (Jami, 1992). The continuous information from the muscle spindles and Golgi tendon organs about the changes in muscle length and tension enables the brain to calculate the body and limb position (Gregory et al., 1987; Jami, 1992; Proske, 2015).

The eyeballs are the most mobile organs of the body and are moved by six pairs of extraocular muscles (EOMs). Because vision is useful only together with the awareness of the direction of viewing, it was postulated (Sherrington, 1918) that, like the skeletal muscles, the EOMs should be endowed with classical proprioceptors for positional information. Surprisingly, the opposite is the case and, with few exceptions, the classical proprioceptor pair is absent in most mammalian species (Maier et al., 1974). Despite the lack of proprioceptors, behavioural and experimental studies have provided evidence that proprioceptive signals from the EOMs reach the brain (Balslev & Miall, 2008; Balslev et al., 2022; Donaldson, 2000; Gauthier et al., 1990; Steinbach & Smith, 1981; Wang et al., 2007). This suggests that the brain uses sensory feedback from EOMs to calculate eye position. The paradox of monitoring EOM activity in the absence of typical proprioceptors seemed to be resolved when a particular nerve specialization termed palisade ending was detected in EOMs of mammals (Dogiel, 1906; Huber, 1900). In fact, for decades there was consensus that palisade endings substitute for muscle spindles and Golgi tendon organs and that they provide eye position information (Alvarado‐Mallart & Pincon‐Raymond, 1979; Billig et al., 1997; Dogiel, 1906; Wang et al., 2007). However, recent studies have called into question the sensory role of palisade endings (Konakci, Streicher, Hoetzenecker, Blumer, et al., 2005; Lienbacher et al., 2011; Zimmermann et al., 2013). This review aims to recapitulate literature including data from our research group on EOM proprioceptors and palisade endings and to consider current knowledge about their structure, molecular characteristics and function.

## 2 MUSCLE SPINDLES IN EXTRAOCULAR MUSCLES

**FIGURE 2:**
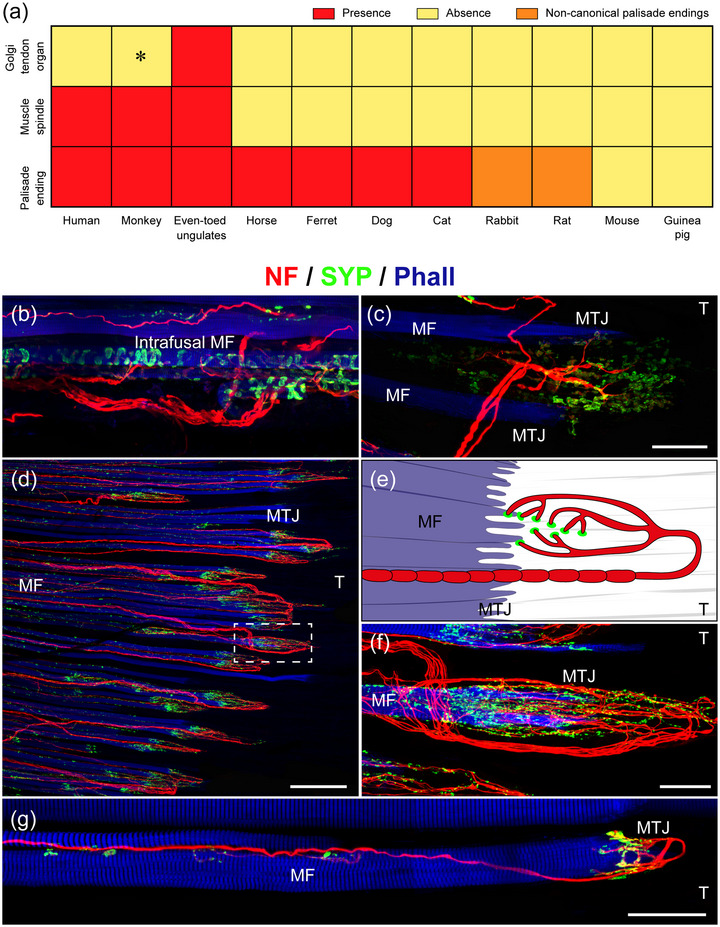
Classical proprioceptors and palisade endings in the EOMs of mammals. (a) Heatmap showing the presence or absence of classical proprioceptors and palisade endings in eye muscles in different mammals. Golgi tendon organs are present in even‐toed ungulates. In monkeys (asterisk), they are present in some, but not all, EOMs. The rest of the species lack Golgi tendon organs. Muscle spindles are only found in humans, monkeys and even‐toed ungulates. Palisade endings are present in all species, except mice and guinea pigs. In rabbits and rats, the palisade endings differ from the canonical palisade endings of higher mammals. (b, c) Visualization of classical proprioceptors in pig EOMs by immunofluorescence. Nerve fibres (red) are labelled with an antibody against neurofilament (NF), and nerve terminal (green) with an antibody against synaptophysin (SYP). Muscle fibres (blue) are counterstained with phalloidin (Phall). (b) The equatorial region of a muscle spindle. The axons (red) spiral around the intrafusal muscle fibres (blue) and establish synaptophysin‐positive contacts (green) on the intrafusal muscle fibres. (c) A Golgi tendon organ at the muscle–tendon junction. The tendon is not labelled and continues to the right of the muscle fibres (blue). The Golgi tendon organ is innervated by a single axon (red) which divides into several branches inside the Golgi tendon organ. Axonal branches establish synaptophysin‐positive nerve terminals (green). MF, muscle fibre; MTJ, muscle–tendon junction; T, tendon. (d–g) Palisade endings in cat EOMs are shown following immunofluorescence staining using the same staining combination as for the muscle spindle and the Golgi tendon organ and in schematic drawing (e). In the fluorescence staining (d, f, g) the tendon is not visible and continues to the right of the muscle fibres (blue). (d) Low magnification image showing palisade endings at the muscle–tendon junction. Palisade endings are formed by nerve fibres (red) which come from the muscle and extend into the tendon. There, they turn back to approach the muscle–tendon junction. By further splitting, axons establish synaptophysin‐positive terminal varicosities (green) around single muscle fibre tips. (e) Schematic drawing of a palisade ending. Terminal varicosities (green) of the palisade ending are found at the level of the tendon (T) and the muscle fibre (MF) tip. (f) The palisade ending from the inset in (d) is shown at high magnification. (g) The nerve fibre (red) that forms a palisade ending establishes multiple synaptophysin‐positive *en grappe* motor terminals (green) along the muscle fibre (blue). (f, g) From the publication ‘Palisade endings have an exocytotic machinery but lack acetylcholine receptors and distinct acetylcholine esterase activity’ (Blumer et al., 2020).

Muscle spindles are rarely found in mammalian EOMs. Except for even‐toed ungulates (pig, sheep, calf and camel) and primates (human and monkey), muscle spindles are absent in the EOMs of mammals (Abuel Atta et al., 1997; Blumer et al., 2001; Harker, 1972; Maier et al., 1974; Rungaldier, Heiligenbrunner et al., 2009; Ruskell, 1990). In pigs, the number of EOM muscle spindles is very high and varies between 148 and 310 muscle spindles per muscle (inferior oblique muscle: 148; superior oblique muscle: 310) (Blumer et al., 2001; Maier et al., 1974). The extremely high number of muscle spindles in the EOMs of ungulates is not equalled in the EOMs of primates. Specifically, between 18 and 34 muscle spindles (18 muscle spindles in the medial rectus and 34 muscle spindles in the inferior rectus muscle) were counted in human EOMs (Lukas et al., 1994), whereas in monkeys only six muscle spindles were counted in the rectus muscles but none in the oblique muscles (Greene & Jampel, 1966). The occurrence of muscle spindles in the EOMs across mammals is shown in Figure 2a.

Detailed structural analyses of EOM spindles were performed in sheep (Harker, 1972; Rungaldier, Heiligenbrunner et al., 2009), pigs (Kubota, 1988), calves (Blumer et al., 2003) and men (Blumer et al., 1999; Lukas et al., 1994; Ruskell, 1989). Only the EOM muscle spindles of even‐toed ungulates (sheep, calf and pig) morphologically conformed to skeletal muscle spindles (Blumer et al., 2003; Harker, 1972; Kubota, 1988; Rungaldier, Heiligenbrunner et al., 2009). In detail, EOM muscle spindles of ungulates have a fusiform shape and a capsule of perineural cells. Inside the spindle, two types of intrafusal muscle fibres with different nuclear arrangements in the equatorial region can be distinguished: nuclear chain fibres, which have a row of centrally arranged nuclei, and nuclear bag fibres, which show a cluster of nuclei. Both intrafusal fibres receive sensory innervation in the equatorial region where axons spiral around the intrafusal fibres. Whether secondary sensory endings, which are common in skeletal muscle spindles and flank the primary sensory endings, are also present in EOM muscle spindles is unclear. Besides this sensory part, EOM muscle spindles in ungulates also have a motor innervation. Specifically, thin motor axons establish motor terminals on intrafusal muscle fibres in the polar region and outside the muscle spindle (Rungaldier, Heiligenbrunner et al., 2009). Figure 2b shows annulospiral nerve terminals in the equatorial region of a muscle spindle from pig EOM.

Several structural particularities were uncovered in the human EOM spindles (Blumer et al., 2006; Bruenech & Ruskell, 2001). Specifically, the muscle spindles are small in diameter and most of them lack equatorial expansion. Additionally, nuclear bag fibres are absent in human EOM spindles, and besides nuclear chain fibres, so‐called anomalous fibres which resembled extrafusal muscle fibres are regularly present. Some intrafusal fibres appear fractured and terminated close to the poles but also at the level of the equatorial region (Blumer et al., 1999; Ruskell, 1989; Bruenech & Ruskell, 2001). All nuclear chain fibres receive annulospiral sensory nerve terminals in the equatorial region, but many anomalous fibres lack sensory innervation (Blumer et al., 1999; Ruskell, 1989). Motor innervation was observed in the polar region of human EOM muscle spindles (Blumer et al., 1999). The structural particularities in human EOM spindles were not only observed in aged persons (Lukas et al., 1994; Ruskell, 1989), but were also present in infants (Blumer et al., 1999; Bruenech & Ruskell, 2001). Thus, the atypical features in human EOM muscle spindles are not attributable to age‐related alterations but might represent a functional specialization.

In mice skeletal muscle spindles, molecular analyses have demonstrated that the annulospiral sensory nerve terminals exhibit characteristics of cholinergic synapses (Zhang et al., 2015, 2014). Specifically, the annulospiral nerve terminals express choline acetyltransferase (ChAT), the synthesizing enzyme of acetylcholine, and are associated with acetylcholine receptors (Zhang et al., 2015, 2014). So far molecular analyses on EOM muscle spindles have only been performed in sheep (Rungaldier, Heiligenbrunner et al., 2009). Different from the skeletal muscle spindles, the annulospiral nerve terminals of EOM muscle spindles do not express ChAT and do not have acetylcholine receptors (Rungaldier, Heiligenbrunner et al., 2009). These findings suggest that skeletal muscle spindles and EOM spindles exhibit differences at the molecular level.

The function of EOM spindles was tested in sheep (Manni et al., 1966). Following stretching of the EOMs, signals which were typical for muscle spindle afferents were recorded in the ipsilateral trigeminal ganglion (Manni et al., 1966). This finding indicates that the ungulate EOM spindles are stretch‐sensitive and register changes in muscle length. Support for the idea that human EOM spindles respond to stretch came from a recent study that used functional magnetic resonance imaging to visualize neuronal activity (Balslev et al., 2022). It was shown that the stretch of the right lateral rectus was associated with the increased neuronal activity of the left oculomotor and left abducens nuclei. The authors (Balslev et al., 2022) have speculated that following muscle stretch, afferents from EOM spindles are transmitted to the EOM motor nuclei at the contralateral side where they evoke activity of the EOM motoneurons. The pathway by which afferents from human EOM spindles reach the brainstem at the contralateral side is unknown.

## 3 GOLGI TENDON ORGANS IN EXTRAOCULAR MUSCLES

Like muscle spindles, Golgi tendon organs are exceptional in mammalian EOMs. They are only present in even‐toed ungulates (sheep (Rungaldier, Heiligenbrunner et al., 2009; Ruskell, 1990), pigs (Blumer et al., 2001), calves (Blumer et al., 2003) and camels (Abuel Atta et al., 1997)) and in primates (monkeys) (Ruskell, 1979). Whereas Golgi tendon organs are numerous in even‐toed ungulates (between 104 and 125 Golgi tendon organs were counted in pig EOMs) (Blumer et al., 2001), they are infrequent in monkeys. That is, some monkey EOMs contain one or two Golgi tendon organs and other muscles none (Ruskell, 1979). In the rest of the mammalian species including humans, Golgi tendon organs are absent. As significant numbers of Golgi tendon organs are exclusively found in the EOMs of even‐toed ungulates, it seems that they are only relevant for this animal group. The occurrence of Golgi tendon organs in EOMs across mammals is shown in Figure 2a.

Golgi tendon organs in the EOMs of even‐toed ungulates have a fusiform shape and a capsule of perineural cells. Typically, a single myelinated nerve fibre enters the Golgi tendon organ and after dividing into several branches, nerve terminals establish intimate contact with the collagen bundles of the Golgi tendon organs (Figure 2c shows a Golgi tendon organ from pig EOM). EOM Golgi tendon organs exhibit some features that are not found in Golgi tendon organs of skeletal muscles (Blumer et al., 2001; Ruskell, 1989). Specifically, EOM Golgi tendon organs have a large fluid‐filled space between the collagen and the capsule, and only one to three muscle fibres are attached outside to the Golgi tendon organ. Additionally, in some cases, up to three muscle fibres enter the Golgi tendon organ, and after entrance, the muscle fibres either terminate in collagen bundles of the Golgi tendon organ or pass through the organ (Blumer et al., 2001).

So far, physiological studies on EOM Golgi tendon organs are missing. Considerations about their possible function are based on anatomical studies. As an analogue to the classical Golgi tendon organ in skeletal muscles, EOM Golgi tendon organs lie in series with the muscle fibres, and it is therefore most likely that they register muscle fibre contraction. Because only a very few muscle fibres are attached to the EOM Golgi tendons organs, they would register the contractions of very few muscle fibres. Such individual monitoring of muscle activity indicates that the brain receives feedback about very delicate eye movements. The effect of muscle fibres passing through the Golgi tendon organs is unclear at the moment.

## 4 PALISADE ENDINGS IN EXTRAOCULAR MUSCLES

At the beginning of the 20th century, Huber (1900), Dogiel (1906) and Tozer and Sherrington (1910) were the first to describe a particular nerve specialization in the EOMs of rabbits, cats and monkeys. This structure was termed the palisade ending, later also known as the innervated myotendinous cylinder. More recent studies in single species (Alvarado‐Mallart & Pincon‐Raymond, 1979; Eberhorn et al., 2005; Lukas et al., 2000; Richmond et al., 1984; Ruskell, 1978; Rungaldier, Pomikal et al., 2009) and our systematic study of 13 mammalian species across six different orders (rodents, lagomorphs, carnivores, perissodactyls, artiodactyls and primates) (Blumer et al., 2016) confirmed that palisade endings are present in most mammals. Specifically, among frontal‐eyed mammals, palisade endings are regularly present in humans (Lukas et al., 2000; Richmond et al., 1984), monkeys (Ruskell, 1978; Tozer & Sherrington, 1910), cats (Alvarado‐Mallart & Pincon‐Raymond, 1979), ferrets (Blumer et al., 2016) and dogs (Rungaldier, Pomikal et al., 2009). Among lateral‐eyed mammals, they are present in even‐toed ungulates (pigs and sheep), odd‐toed ungulates (horses), lagomorphs (rabbits) and rats (Eberhorn et al., 2005), and are absent only in mice and guinea pigs (Blumer et al., 2016). Counts revealed that the number of palisade endings is higher in frontal‐eyed species than in those lateral‐eyed species equipped with palisade endings (Blumer et al., 2016). The wide distribution of palisade endings across mammals suggests that they might substitute classical proprioceptors although they might be more relevant for the frontal‐eyed than the lateral‐eyed species. The occurrence of palisade endings in EOMs across mammals is shown in Figure 2a.

Palisade endings are present in all rectus EOMs although, in frontal‐eyed species, many more palisade endings are found in the medial rectus than in the other rectus muscles (Blumer et al., 2016, 2017; Lienbacher et al., 2018). In cats, monkeys, and humans, palisade endings were also found in the oblique EOMs (Alvarado‐Mallart & Pincon‐Raymond, 1979; Lukas et al., 2000; Lienbacher et al., 2011). As demonstrated in a frontal‐eyed species (cat), palisade endings are immature at birth and develop during the first 3 months of life in a muscle‐specific sequence (Blumer et al., 2017). The proper development of palisade endings relies on eye movements in the early postnatal period as the block of eye movements by botulinum neurotoxin substantially delays the palisade ending maturation (Carrero‐Rojas et al., 2022).

Palisade endings are exuberant axonal terminations at the muscle–tendon junction of the global layer (inner layer) of the EOMs (Figure 2d, f and g show palisade endings in cat EOMs). So far, no palisade endings have been found in the orbital layer (outer layer) of the EOMs which faces the bony wall of the orbit. Palisade endings are formed by myelinated nerve fibres that come from the muscle and extend into the tendon. There, the axons make a u‐shaped turn to approach the muscle–tendon junction. By further branching, axons establish terminal varicosities at the level of the tendon and around single muscle fibre tips (Figure 2e and f). Interestingly, palisade endings in rabbits and rats differ from the palisade endings in higher mammals. Specifically, palisade endings in rabbits and rats lack terminal varicosities at the tendon level and only terminal varicosities around the muscle fibre tips are found (Blumer et al., 2016; Eberhorn et al., 2005).

The muscle fibres associated with palisade endings possess several *en grappe* terminals along their length. They are consequently classified as multiply innervated muscle fibres (MIFs) (Alvarado‐Mallart & Pincon‐Raymond, 1979; Ruskell, 1978). This is opposed to the singly innervated muscle fibres (SIFs) in EOMs which correspond to the classical skeletal muscle fibres and receive a single and large motor endplate. SIFs and MIFs are found in both the global and the orbital layer of the EOMs (Mayr et al., 1975). The MIFs exhibit a non‐twitch form of contraction and the cell bodies of MIF motoneurons are located at the border of the EOM nuclei although the specific pattern of arrangement is different across species (Bohlen et al., 2016; Buttner Ennever et al., 2001; Carrero‐Rojas et al., 2021; Hernandez et al., 2019).

Although the sensory innervation of EOMs is a contentious issue, there has been a consensus for decades that palisade endings are sensory structures and have proprioceptive function. The reason is that palisade endings lie in series with muscle fibres and it has been assumed that they are in an ideal position to register muscle fibre contraction (Alvarado‐Mallart & Pincon‐Raymond, 1979). Support for the idea that palisade endings are proprioceptors came from fine structural analyses which were carried out almost simultaneously in monkeys (Ruskell, 1978) and cats (Alvarado‐Mallart & Pincon‐Raymond, 1979). In both animal species, the terminal varicosities of palisade endings establish contact with the collagen fibrils of the tendon. Such neurotendinous contacts resemble sensory nerve terminals of Golgi tendon organs. As opposed to the common neurotendinous contacts, few terminal varicosities of palisade endings establish contact with the muscle fibres. These neuromuscular contacts lack a basal lamina in the synaptic cleft, a feature typical for sensory nerve terminals in muscle spindles. Altogether, morphological studies revealed that the palisade endings exhibit structural characteristics in common with the classical proprioceptors. Findings in a single neuronal tracing experiment reinforced the sensory nature of palisade endings (Billig et al., 1997). Following the injection of neuronal tracer into the trigeminal ganglion, three kinds of tracer‐labelled nerve terminations were found in the EOMs, one type resembling the palisade ending (Billig et al., 1997). Because the trigeminal ganglion is a sensory ganglion, this finding provided direct evidence that palisade endings are sensory structures.

Indirect indications that palisade endings are sensory came from other studies (Steinbach & Smith, 1981; Steinbach et al., 1987; Wang et al., 2007). Specifically, a single physiological experiment in monkeys demonstrated that the neuronal activity in area 3 of the somatosensory cortex increased when the animal moved its eyes (Wang et al., 2007). This finding proved that the EOMs are endowed with sensory organs to register muscle contraction and to send this information to the brain. In monkey EOMs, muscle spindles and Golgi tendon organs are rare or absent whereas palisade endings occur in very high numbers (Ruskell, 1978). Consequently, the palisade ending is the only possible candidate to transmit eye position information to the brain's somatosensory cortex whereby the exact route for this information is unclear, yet. Clinical studies in squint patients (Steinbach & Smith, 1981; Steinbach et al., 1987) showed that patients undergoing marginal myotomy for correction of the eye position exhibited deficits in spatial perception. Because palisade endings were partly or completely removed by the marginal myotomy, it was assumed that the spatial perception deficits were a result of missing sensory feedback from palisade endings after surgery (Steinbach & Smith, 1981; Steinbach et al., 1987). In this context, it is important to note that the spatial perception deficits in patients were observed a short time after the operation (between 7 and 48 h), and it would be of interest to know if the deficits disappear or are present over a longer period of time.

In 2005, the molecular phenotype of the palisade endings was uncovered by our research group (Konakci, Streicher, Hoetzenecker, Blumer, et al., 2005; Konakci, Streicher, Hoetzenecker, Haberl, et al., 2005). Because the terminal varicosities of palisade endings are full of clear vesicles which are typical for cholinergic motor terminals, we hypothesized that palisade endings are potentially cholinergic organs. We tested different cholinergic markers in palisade endings including antibodies against ChAT, vesicular acetylcholine transporter (VAChT) and choline transporter (ChT). We observed that, like motor terminals, palisade endings in cats and monkeys were immunoreactive for ChAT (Konakci, Streicher, Hoetzenecker, Blumer et al., 2005; Konakci, Streicher, Hoetzenecker, Haberl, et al., 2005). Additional experiments in monkeys revealed that palisade endings also expressed VAChT and ChT. Unfortunately, the antibody against VAChT and ChT did not work in cats. Later, the cholinergic phenotype of palisade endings was confirmed in other mammals including humans (Rungaldier, Heiligenbrunner, et al., 2009; Rungaldier, Pomikal, et al., 2009; Lienbacher et al., 2018a, 2019). The novel insight into the molecular identity of palisade endings reopened the debate about the functional significance of this EOM‐specific structure.

That palisade endings exhibit a cholinergic phenotype was not easily compatible with the previous notion that they originate in the sensory trigeminal ganglion (Billig et al., 1997). Doubts that the source of the palisade endings lies in the trigeminal ganglion came from two older studies (Sas & Scháb, 1952; Tozer & Sherrington, 1910). Specifically, following a lesion of the oculomotor nucleus or severance of the cranial motor nerves (oculomotor, trochlear and abducens nerve) innervating the EOMs, degeneration of the palisade endings was observed. These findings suggested that palisade endings originate in the EOM motor nuclei and not in the trigeminal ganglion. Because of these discrepancies regarding the source of the palisade endings, the central connections of the palisade endings were reinvestigated. These investigations were independently carried out by the research group of Prof. Horn and our research group. Following the injection of neuronal tracer into the EOM motor nuclei, tracer‐labeled palisade endings were found in the EOMs of monkeys (Lienbacher et al., 2011; Zimmermann et al., 2011) and cats (Zimmermann et al., 2013). Additionally, it was observed that the axons forming palisade endings establish multiple motor terminals along the muscle fibres associated with palisade endings (Blumer et. al. et al., 2020; Zimmermann et al., 2013). These findings confirmed that palisade endings originate in the EOM motor nuclei, most likely, from the MIF motor neurons of which they are the peripheral expansions (Figure 2g).

Because molecular and neuronal tracing studies suggested that palisade ending are effectors, we tested whether palisade endings have exocytotic machinery for neurotransmitter (acetylcholine) release (Blumer et al., 2020). Key proteins in neuronal exocytosis are SNAP25, synaptotagmin, syntaxin, complexin and synaptobrevin (Fernandez‐Chacon et al., 2001; Sutton et al., 1998). Using immunohistochemistry, we confirmed that these exocytotic proteins are also expressed in the palisade endings. This indicates that palisade endings have the molecular prerequisite for neurotransmitter (acetylcholine) release. Surprisingly, other key features of cholinergic synapses were not found in palisade endings. Specifically, the enzyme acetylcholine esterase (AChE), which degrades acetylcholine to terminate neuronal transmission, was absent in most palisade endings and in others it was only present at low concentrations as demonstrated by anti‐AChE immunohistochemistry (Blumer et al., 2020). Additionally, we did not find receptors for acetylcholine on muscle fibres associated with palisade endings as demonstrated by the absence of α‐bungarotoxin (a snake venom that binds to acetylcholine receptors) and anti‐acetylcholine receptor signals. Exceptions are only the palisade endings of rabbits and rats, where, different from the canonical palisade endings of higher mammals, acetylcholine receptors were found (Blumer et al., 2016).

## 5 FUNCTIONAL CONSIDERATIONS ON PALISADE ENDINGS

Neuronal tracing and immunohistochemical studies have shown that palisade endings are the peripheral expansion of MIF motor axons (Blumer et al., 2020, 2017; Zimmermann et al., 2013). Because of this structural unit, the excitation of MIF motor axons would induce neurotransmitter release at the site both of the *en grappe* motor terminals and of the palisade endings. *En grappe* motor terminals are endowed with acetylcholine receptors and neurotransmitter release would induce contraction of the muscle fibre body, whereas palisade endings lacking acetylcholine receptors would exert no effect on the terminal portion of the muscle fibre opposite to the palisade ending. Moreover, due to the low concentration or absence of AChE, the fast removal of acetylcholine is insufficient. Consequently, the effect of acetylcholine release from palisade endings is difficult to predict, but is possible that acetylcholine set free from palisade endings binds to hitherto unknown receptors or serves as a receptor‐independent, modulatory function.

Although the functional significance of palisade endings is still debated, there are indications that the palisade endings might be relevant for convergence eye movements, which are crucial for focusing on close objects or performing near‐work activities. During convergence eye movements, both eyes move inwards requiring the coordinated activity of both medial rectus muscles. It has been shown in frontal‐eyed species that many more palisade endings are present in the medial rectus muscle than in the other rectus muscles (Blumer et al., 2016; Lienbacher et al., 2018). Moreover, in a frontal‐eyed species (cat) it has been demonstrated that palisade endings are fully developed significantly earlier in the medial rectus muscle than in the other rectus muscles (Blumer et al., 2017). Finally, in primates (monkey and man), many palisade endings of the medial rectus express calretinin, and it is hypothesized that the calretinin‐positive palisade endings represent a specialized, probably more excitable type of palisade ending required for convergence eye movements (Lienbacher et al., 2018, 2019).

## 6 CONCLUSION

The present review has shown that the occurrence of classical proprioceptors in EOMs varies widely across mammalian species. Exclusively in the EOMs of even‐toed ungulates, muscle spindles and Golgi tendon organs are present. In the EOMs of primates (monkey and man), only muscle spindles with a simplified morphology are present, and in the remainder of species, muscle spindles and Golgi tendon organs are lacking at all. Because the classical proprioceptor pair is only present in the EOMs of even‐toed ungulates, only this animal group is equipped with a traditional proprioceptive feedback system to monitor the EOM activity. Whether this correlates with any eye movement properties in even‐toed ungulates is currently unknown. Most mammalian species possess palisade endings in the EOMs, and there was an agreement for many years that palisade endings substitute for classical proprioceptors and provide eye position information. Today we are faced with the fact that palisade endings combine sensory as well as motor characteristics. Because of this ambiguity, it is challenging to sort out the function of palisade endings. In the future, further molecular profiling and electrophysiological experiments are needed to clarify the functional role of palisade endings.

## AUTHOR CONTRIBUTIONS

Roland Blumer conceptualized and wrote the manuscript. All authors made critical revisions and approved the final version of the manuscript. All authors agree to be accountable for all aspects of the work in ensuring that questions related to the accuracy or integrity of any part of the work are appropriately investigated and resolved. All persons designated as authors qualify for authorship, and all those who qualify for authorship are listed.

## CONFLICT OF INTEREST

None declared.
